# Longitudinal associations of sedentary behavior and physical activity with body composition in colorectal cancer survivors up to 2 years post treatment

**DOI:** 10.1007/s00432-022-04267-9

**Published:** 2022-08-30

**Authors:** Marlou-Floor Kenkhuis, Mo Klingestijn, Anne-Marie Fanshawe, Stéphanie O. Breukink, Maryska L. G. Janssen-Heijnen, Eric T. P. Keulen, Sabina Rinaldi, Paolo Vineis, Marc J. Gunter, Michael F. Leitzmann, Augustin Scalbert, Matty P. Weijenberg, Martijn J. L. Bours, Eline H. van Roekel

**Affiliations:** 1 Department of Epidemiology, GROW School for Oncology and Reproduction, Maastricht University, P.O. BOX 616, 6200 MD Maastricht, The Netherlands; 2 Department of Epidemiology, GROW School for Oncology and Reproduction, Maastricht University as Part of an Internship, Maastricht University, Maastricht, The Netherlands; 3 Department of Surgery, GROW School for Oncology and Reproduction, NUTRIM School of Nutrition and Translational Research in Metabolism, Maastricht University Medical Centre+, Maastricht, The Netherlands; 4 Department of Clinical Epidemiology, Viecuri Medical Center, Venlo, The Netherlands; 5 Department of Internal Medicine and Gastroenterology, Zuyderland Medical Centre, Sittard-Geleen, The Netherlands; 6 Nutrition and Metabolism Branch, International Agency for Research On Cancer (IARC-WHO), Lyon, France; 7 MRC Centre for Environment and Health, School of Public Health, Imperial College, London, UK; 8 Italian Institute of Technology, Genoa, Italy; 9 Department of Epidemiology and Preventive Medicine, University of Regensburg, Regensburg, Germany

**Keywords:** Physical activity, Sedentary behavior, Body composition, Colorectal cancer survivors, Fatigue

## Abstract

**Purpose:**

We investigated longitudinal associations of sedentary behavior, light-intensity physical activity (LPA) and moderate-to-vigorous physical activity (MVPA) with body composition in colorectal cancer (CRC) survivors, between 6 weeks and 24 months post treatment. In addition, we explored whether body composition mediated associations of sedentary behavior and MVPA with fatigue.

**Methods:**

A prospective cohort study was conducted in 459 stage I–III CRC patients recruited at diagnosis. Measurements were performed of accelerometer-assessed sedentary time (hours/day), self-reported LPA and MVPA (hours/week), anthropometric assessment of body mass index (BMI), waist circumference and fat percentage (measures of adiposity), and muscle circumference and handgrip strength (measures of muscle mass/function) repeated at 6 weeks, and 6, 12 and 24 months post treatment. Longitudinal associations of sedentary time and physical activity with body composition were analyzed using confounder-adjusted linear mixed models. Mediation analyses were performed to explore the role of body mass index (BMI) and handgrip strength as mediators in associations of sedentary time and MVPA with fatigue.

**Results:**

Less sedentary time and LPA were, independent of MVPA, longitudinally associated with increased handgrip strength, but not with measures of adiposity. More MVPA was associated with increased adiposity and increased handgrip strength. Higher BMI partly mediated associations between higher sedentary time and more fatigue.

**Conclusion:**

Within the first two years after CRC treatment, changes in sedentary behavior, physical activity and body composition are interrelated and associated with fatigue. Intervention studies are warranted to investigate causality.

**Trial registration:**

The EnCoRe study is registered at trialregister.nl as NL6904 (former ID: NTR7099).

**Supplementary Information:**

The online version contains supplementary material available at 10.1007/s00432-022-04267-9.

## Introduction

Colorectal cancer (CRC) is currently the third most commonly diagnosed cancer worldwide (Sung et al. 2020). The aging and growth of the population, as well as an increasing trend in unhealthy lifestyle behaviors have resulted in a marked rise in global CRC incidence. At the same time, advancements in treatment and earlier detection as a result of screening programs have led to an upward trend in survival after CRC (Bray et al. 2013; Ferlay et al. 2015; Parry et al. 2011). Due to the increasing incidence and improved survival rate, the total population of CRC survivors is expected to rise in the upcoming years. CRC survivors often report long-term side effects of cancer and/or its treatment, such as fatigue (Hofman et al. 2007), which impede health-related quality of life (HRQoL) (Jansen et al. 2011).

Previous research consistently shows that obesity is linked to a higher risk of CRC (Bianchini et al. 2002; Haydon et al. 2006), leading to a high prevalence of obesity among CRC survivors (Veen et al. 2019; Kenkhuis et al. 2021a). Although we previously reported that increased BMI was associated with less fatigue in the first two years after treatment (Kenkhuis et al. 2021a), most studies in long-term (> 5 years) CRC survivors found that higher BMI was associated with more fatigue (Vissers et al. 2017; Neefjes et al. 2017). Due to the often high prevalence of obesity observed in CRC survivors, it is important to find a way to improve their body composition and consequently decrease fatigue and improve HRQoL.

A meta-analysis of randomized controlled trials (RCTs) on the effects of moderate-to-vigorous physical activity (MVPA) on BMI and cancer-related fatigue in survivors who had completed their main cancer treatment showed that increased MVPA can slightly reduce BMI and reduce cancer-related fatigue (Fong et al. 2012). In the specific population of CRC survivors, two RCTs assessed whether exercise training can improve body composition quantified using dual energy X-ray absorptiometry (Devin et al. 2016; Brown et al. 2017). One study, among 39 stage I–III CRC survivors, showed a decrease in visceral adipose tissue after aerobic exercise (Brown et al. 2017). The other RCT of 47 CRC survivors showed that high intensity aerobic training increases whole-body lean mass and decreases whole-body adipose tissue (Devin et al. 2016). These studies focused on MVPA, which comprised activities that have a greater intensity than 3 metabolic equivalent of tasks (METs), including for example brisk walking or cycling (Ainsworth et al. 1993). However, MVPA comprises less than 2% of all physical activity during waking hours among cancer survivors above 60 years old (Lynch et al. 2013).

Few studies have examined behaviors that account for much larger proportions of activities during waking hours, such as sedentary behavior (± 68%) or light-intensity physical activity (LPA, ± 31%) (Lynch et al. 2013). Sedentary behavior is defined as any behavior characterized by an energy expenditure ≤ 1.5 METs while in a sitting or reclining posture (Tremblay et al. 2017; Bames et al. 2012), and LPA comprised activities that have movement intensities between 1.5 and 3 METs (Wendel-Vos et al. 2003). According to our knowledge, one cross-sectional study and one prospective study have examined how sedentary behavior or LPA are associated with body composition in CRC survivors (Lynch et al. 2016; Wijndaele et al. 2009). Both studies in colon cancer survivors showed that more sedentary behavior was associated with higher BMI and more LPA and MVPA with lower BMI (Lynch et al. 2016; Wijndaele et al. 2009). However, one of these studies was cross-sectional (Lynch et al. 2016), and the other one was using television time (Wijndaele et al. 2009); therefore, there is a need for longitudinal studies to assess the relationship of accelerometer-assessed sedentary behavior and physical activity, including both MVPA and LPA, with a comprehensive spectrum of measures of body composition outcomes after CRC. In addition, because we previously reported that body composition is associated with both physical activity and fatigue (Kenkhuis et al. 2021a, 2021b), it is important to investigate whether body composition may play a mediating role in the association between physical activity and fatigue.

Hence, we aimed to investigate longitudinal associations of self-reported MVPA and LPA, and accelerometer-assessed sedentary behavior with anthropometric measures of adiposity, muscle mass and muscle function in CRC survivors, between 6 weeks and 24 months post treatment. Furthermore, we aimed to gain insight into potential mechanisms through which physical activity may influence fatigue in CRC survivors by exploring the potential role of body composition as a mediator.

## Methods

### Study design

The current study is embedded within the Energy for life after ColoRectal cancer (EnCoRe) study. This is a multi-center prospective cohort study initiated in 2012, in which patients diagnosed with stage I–III CRC at three hospitals in the south-eastern region of the Netherlands (Maastricht University Medical Centre + , VieCuri Medical Centre, and Zuyderland Medical Centre) are included. Eligible for participation were men and women above the age of 18, diagnosed with stage I, II, or III CRC. Exclusion criteria were stage IV CRC, inability to understand and speak Dutch, residence outside of the Netherlands, or the presence of co-morbidities hindering successful participation (e.g., Alzheimer and visibility/hearing disorder) (Roekel et al. 2014). Data available up to July 16, 2018 were used for the present analyses, including data from participants included at diagnosis (*n* = 459) and followed up with repeated measurements at 6 weeks (*n* = 396), 6 months (*n* = 348), 12 months (*n* = 287), and 24 months post treatment (*n* = 208). The main reason for the decrease in numbers as follow-up time increases was due to participants with data available at diagnosis not having reached some of the subsequent follow-up points on July 16, 2018. Participation rate at diagnosis was 45% and follow-up participation rate was above 91% at all post-treatment follow-up time points. A detailed flow-diagram was previously published by Kenkhuis et al. (2021a). The EnCoRe study has been approved by the Medical Ethics Committee of the University Hospital Maastricht and Maastricht University (Netherlands Trial Register number NL6904) (Roekel et al. 2014). All participants provided written consent.

### Sedentary behavior and physical activity

Total sedentary time, prolonged sedentary time (in hours/day) and standing (in hours/day) were objectively measured at all post-treatment time points using the tri-axial accelerometer-based MOX activity monitor (MMOXX1, upgraded version of the CAM; Maastricht Instruments B.V., NL) (Annegarn et al. 2011). Participants wore the device for seven consecutive days, 24 h/day. This device is attached to the anterior upper thigh 10 cm above the knee using a skin-friendly plaster. The placement on the upper thigh was chosen since it can measure leg position and body posture, and thereby accurately distinguish sitting and lying from upright positions (Annegarn et al. 2011; Berendsen et al. 2014). The device showed a good validity for posture classification (kappa = 0.95), and walking intensity (Spearman’s *r* = 0.96) in healthy individuals (Berendsen et al. 2014). Prolonged sedentary time (hours/day) is derived by summing total daily time accrued in sedentary bouts with a duration of at least 30 min (Chastin and Granat 2010; Stephens et al. 2014). Although the device also measures total physical activity (all activities with an energy expenditure > 1.5 METs), the monitor unfortunately has limited reproducibility to distinguish between light and moderate-to-vigorous physical activity (Berendsen et al. 2014). Therefore, LPA and MVPA were measured by means of a validated questionnaire, as described below. Accelerometer data were deemed valid with ≥ 10 h of waking wear time per day; only participants with ≥ 4 valid days were included in analyses.

Self-reported time spent in LPA and MVPA was measured at all time points (including at diagnosis) using the Short QUestionnaire to ASsess Health-enhancing physical activity (SQUASH) (Wendel-Vos et al. 2003). The SQUASH assesses the intensity, duration and frequency of activities in the previous week, including commuting, work, household and leisure time activities. Activities were categorized according to intensity based on METs (Ainsworth et al. 1993). LPA comprised activities with an intensity below 3 METs (Wendel-Vos et al. 2003) and all activities ≥ 3 METs were categorized as MVPA (Ainsworth et al. 1993). The SQUASH was shown to be fairly reliable (test–retest: Spearman’s ρ 0.57–0.58 (Wendel-Vos et al. 2003; Wagenmakers et al. 2008)). Relative validity, in comparison to accelerometer data, was found to be comparable with other physical activity questionnaires (Spearman ρ 0.20 for light-intensity physical activity; ρ 0.40 for moderate-intensity activity; ρ 0.35 for vigorous-intensity activity) (Wagenmakers et al. 2008).

### Body composition

In accordance with standard operating procedures, trained personnel conducted extensive anthropometric measurements at all time points. Body weight (to the nearest 0.1 kg) was measured in light clothing without shoes using a portable weighing scale (Seca Ltd, Birmingham, UK, electronic scale type 861). Body height (to the nearest 0.1 cm) was measured in duplicate at diagnosis with a portable stadiometer, with the participant standing barefoot against a wall. BMI was calculated as weight (kg) divided by squared mean height (m^2^). BMI was categorized according to the WHO criteria as normal weight (18.5 ≤ BMI < 25 kg/m^2^), overweight (25 ≤ BMI < 30 kg/m^2^), or obesity (BMI ≥ 30 kg/m^2^) (World Health Organization 2011).

As an estimate of visceral adiposity, waist circumference (to the nearest 0.1 cm) was measured with a circumeter (type: 05,335, Premed) midway between the lower rib margin and the ileac crest. The average of duplicate measurements was used for analysis.

Triplicate measurements of skinfold thickness (to the nearest 0.2 mm) was measured at the dominant side of the body using Holtain skinfold calipers (Lohman et al. 1988) (range 0.00–50.00 mm) at the following sites: triceps; biceps; subscapular; and supra-iliac. The sum of median values of the measurements at each of the four skinfolds was used to calculate body fat percentage based on the Durnin–Womersley calculations with the Siri equation (Durnin and Womersley 1974). BMI, waist circumference and body fat were used as measures of adiposity.

Mid-upper arm circumference (MUAC, to the nearest 0.1 cm) of the dominant arm was measured in duplicate with a circumeter at a point midway between the acromion process and the olecranon process (Gurney and Jelliffe 1973). Mid-upper arm muscle circumference (MUAMC) was calculated based on the mean MUAC and the median triceps skinfold thickness using the standard formula: MUAMC = MUAC – (3.1415 × triceps skinfold thickness) (Frisancho 1981).

Isometric handgrip strength was assessed as a proxy of overall muscle strength and function (Lauretani et al. 2003; Cruz-Jentoft et al. 2010). Measurement of maximum handgrip strength (to the nearest kg) was performed with the dominant hand using a handheld dynamometer, with the participant in the seated position and the elbow flexed at 90°. The participant was instructed to squeeze the handle as hard as possible for 3–5 s. The measurement was repeated after a brief recovery period, and the highest value was used for further analysis. The MUAMC and handgrip strength were used as measures of muscle mass and functioning, respectively.

### Fatigue

Data on fatigue were self-assessed using the well-validated and reliable European Organization for Research and Treatment of Cancer Quality of Life Questionnaire C30 (EORTC QLQ-C30) version 3.0 (Aaronson et al. 1993), as well as the validated comprehensive multidimensional Checklist Individual Strength (CIS) (Vercoulen et al. 1996; Servaes et al. 2001). The fatigue symptom scale from the EORTC QLQ-C30 was linearly transformed to a 0–100 scale. The CIS is a 20-item questionnaire, with each item being scored on a 7-point Likert scale and consisting of four subscales: subjective fatigue (range in score: 8–56), concentration problems (5–35), reduced motivation (4–28), and activity-related fatigue (3–21). The total fatigue score (range: 20–140) was obtained by summing all item scores. Higher scores on all scales represent more fatigue. The total fatigue score and activity-related fatigue subscale were included in the current mediation analysis because we expected physical activity and body composition to be associated with the physical dimension of fatigue (Kenkhuis et al. 2021a, 2021b).

### Lifestyle, clinical, sociodemographic factors

Age, sex, and clinical information (i.e., cancer stage, surgery/chemotherapy/radiotherapy treatment, and tumor site) were retrieved from medical records. Self-reported data were collected on other factors, including current smoking status at all time points and highest attained education level at diagnosis. Comorbidities were assessed with the Self-Administered Comorbidity Questionnaire at all post-treatment time points (Sangha et al. 2003). Total dietary energy intake was measured through 7-day food diaries collected at each post-treatment time point (Breedveld-Peters et al. 2018).

### Statistical analysis

Descriptive analyses were performed for sociodemographic, clinical characteristics, sedentary behavior, standing, physical activity and anthropometric measures. Normally distributed continuous variables were described as means and standard deviations (SD) and non-normally distributed variables as medians and interquartile ranges.

### Primary analyses: longitudinal associations

We applied linear mixed model regression to analyze longitudinal associations of sedentary behavior, standing, LPA, and MVPA with anthropometric measures, using data collected between 6 weeks and 24 months post treatment. Each of the exposure variables of interest, i.e., total sedentary time (per 2 h/day), prolonged sedentary time (per 2 h/day), standing (per hour/day), LPA (per 8 h/day), and MVPA (per 150 min/week) were analyzed in separate models as a continuous exposure variables and separate models were run for each of the anthropometric measures as continuous outcomes (BMI, waist circumference, body fat percentage, MUAMC, and handgrip strength). A random intercept for each subject was added to all models. The use of random slopes was tested with a likelihood-ratio test; when the model fit improved statistically significantly random slopes were added.

Based on previous literature on sedentary behavior, physical activity and body composition in CRC survivors and the use of causal diagrams, we adjusted regression models for an a priori defined set of relevant confounders, which contained fixed time-invariant confounders including sex (male, female), age (years at enrollment), chemotherapy (yes, no), MVPA at diagnosis, and the measurement of the anthropometric measure at diagnosis, as well as time-variant confounders measured at all post-treatment time points including time since end of treatment (weeks), number of co-morbidities (0,1, ≥ 2), and total energy intake (kcal/day). To assess independent associations of sedentary behavior (both total sedentary time and prolonged sedentary time) and MVPA with body composition outcomes, models for sedentary behavior were adjusted for MVPA and vice versa, and LPA and standing were adjusted for MVPA. In addition, for the models including accelerometer-assessed variables (total sedentary time, prolonged sedentary time, and standing time), adjustment for waking wear time (hours/day) was done by including this time variable as an additional covariate. We further applied the 10% change-in-estimate method for assessing an additional set of potential confounders: education level (low, middle, high), received radiotherapy (yes, no), tumor site (colon, rectum), and smoking (current, former, never). None of the variables led to > 10% change in beta estimates and were, therefore, not included in the main model. Inter- and intra-individual associations were disaggregated by adding centered person-mean values to the model to estimate inter-individual associations (i.e., due to differences in physical activity or sedentary behavior between individuals), and individual deviations at each time point from the person-mean value to estimate intra-individual associations (i.e., due to changes in physical activity or sedentary behavior within individuals) (Twisk and Vente 2019).

To obtain insight into the possible direction of longitudinal associations, we also performed time-lag analyses. In these analyses, sedentary behavior, standing and physical activity at earlier time points were coupled with body composition variables at subsequent time points to simulate a more natural direction of association.

All descriptive and mixed model analyses were performed using Stata version 14 with statistical significance set at p < 0.05 (two-sided).

### Secondary analyses: mediation analysis

Mediation analyses were conducted to analyze whether body composition 6 months after treatment was involved as a mediator in the association of total sedentary time and MVPA at 6 weeks (exposure) with fatigue at 12 months post treatment (outcome). In addition, similar mediation analyses were conducted using total sedentary time and MVPA at 6 months (exposure), body composition at 12 months post treatment (mediator), and fatigue at 24 months post treatment (outcome).

We used the PROCESS analytic tool developed by Hayes to assess whether both BMI and handgrip strength were mediators in the associations of total sedentary time and MVPA with fatigue. These analyses were based on multiple linear regression path analyses (Hayes 2017). Two paths were separated in the mediation analyses. First, the path from total sedentary time and MVPA to fatigue, independent of body composition (i.e., the direct association). Second, the path from total sedentary time and MVPA to fatigue that passes through potential mediating variables of body composition (both BMI and handgrip strength), which is referred to as the specific indirect association. The sum of the specific indirect association through BMI and the specific indirect association through handgrip strength is referred to as the total indirect association. The sum of the direct and total indirect associations is referred to as the total association. Model 4 of the PROCESS macro version 3.5 for SPSS was used to assess specific indirect, total indirect, direct, and total associations of total sedentary time and MVPA with fatigue. A 95% percentile bootstrap confidence interval for the indirect effect using 10,000 bootstrap samples was generated.

As a sensitivity analysis, the sample size of the 6 months to 24 months mediation analysis (*n* = 170) was also used for the mediation analysis from 6 weeks to 12 months.

## Results

Baseline characteristics are reported at all post-treatment time points (Table 1). A total of 270 males (68%) and 126 females (33%) were included who were on average 67.0 years of age (SD = 9.1). Most participants (*n* = 276, 70%) were overweight or obese (BMI ≥ 25 kg/m^2^). A total of 102 (26%) participants reported having one comorbidity and 202 (51%) reported having two or more co-morbidities. Participants were more often diagnosed with colon than rectum cancer (63% vs. 37%, respectively), and 124 (31%) were stage I, 100 (25%) stage II and 172 (43%) stage III. Received treatments were chemotherapy (39%), radiotherapy (26%) and/or surgery (89%). Sedentary behavior decreased, whereas standing, LPA, and MVPA increased from 6 weeks up to 24 months post treatment. With regard to anthropometric measures, all body composition measures followed similar trends, decreasing from diagnosis to 6 weeks and then increasing up to 24 months post treatment. Changes over time for both the exposures and the outcomes have been extensively described in previous publications (Kenkhuis et al. 2021a, 2021b; Roekel et al. 2020).

**Table 1:** Demographic, lifestyle, and clinical characteristics of colorectal cancer survivors at all time points

	Diagnosis (*n* = 459)	6 weeks post-treatment (*n* = 396)	6 months post-treatment (*n* = 348)	12 months post-treatment (*n* = 287)	24 months post-treatment (*n* = 208)
Sex (male) [*n* (%)]	303 (66.0)	270 (68.2)	236 (67.8)	196 (68.3)	142 (68.3)
Age (years) [mean (SD)]	66.9 (9.1)	67.0 (9.1)	67.2 (9.23)	67.4 (9.2)	68.1 (9.2)
Education [*n* (%)]					
Low	130 (29.0)	107 (27.1)	91 (26.2)	73 (25.5)	45 (21.6)
Medium	168 (37.4)	149 (37.7)	137 (39.5)	114 (39.9)	89 (42.8)
High	151 (33.6)	139 (35.2)	119 (34.3)	99 (34.6)	74 (34.6)
Comorbidities [*n* (%)					
0 co-morbidities	–	91 (23.0)	88 (25.4)	71 (25.1)	46 (22.6)
1 comorbidity	–	102 (25.8)	87 (25.1)	64 (22.6)	49 (24.0)
≥ 2 co-morbidities	–	202 (51.1)	172 (49.6)	148 (52.3)	109 (53.4)
Smoking [*n* (%)]					
Never	139 (31.0)	118 (30.5)	98 (28.7)	76 (27.6)	57 (29.1)
Former	255 (56.8)	235 (60.7)	213 (62.5)	172 (62.6)	120 (61.2)
Current	55 (12.3)	34 (8.8)	30 (8.8)	27 (9.8)	19 (9.7)
Body mass index, kg/m^2^ [mean (SD)]	28.3 (4.7)	27.8 (4.6)	28.3 (4.7)	28.7 (4.8)	28.3 (4.6)
Underweight: < 18.5	2 (0.4)	2 (0.5)	0 (0.0)	1 (0.4)	1 (0.5)
Healthy weight: 18.5–24.9	111 (24.3)	117 (29.6)	90 (25.9)	62 (21.9)	49 (24.0)
Overweight: 25–29.9	201 (44.0)	173 (43.8)	151 (43.5)	130 (45.9)	85 (41.7)
Obese: ≥ 30	143 (31.3)	103 (26.1)	106 (30.6)	90 (31.8)	69 (33.8)
Waist circumference, cm [mean (SD)]	101.5 (13.6)	100.1 (12.9)	101.3 (13.4)	102.3 (13.2)	101.7 (13.1)
Fat percentage, % [mean (SD)]	33.6 (6.4)	32.9 (6.4)	33.4 (6.2)	33.9 (6.3)	33.6 (6.3)
MUAMC, mm [mean (SD)]	249.1 (29.6)	247.7 (28.1)	250.8 (29.6)	253.1 (28.0)	253.7 (29.4)
Handgrip strength, kg [mean (SD)]	38.1 (12.0)	36.3 (11.7)	37.0 (11.8)	37.5 (12.4)	36.9 (12.4)
Total sedentary time, hours/week [mean (SD)]		10.8 (1.8)	10.1 (1.5)	10.2 (1.5)	10.2 (1.4)
Prolonged sedentary time. hours/week [mean (SD)]	–	5.3 (2.7)	4.2 (1.9)	4.4 (1.9)	4.5 (1.9)
Light physical activity, minutes/week [median (IQR)]	660 (1350)	450 (930)	630 (1080)	630 (1080)	630 (1080)
Moderate-to-vigorous physical activity. minutes/week [median (IQR)]	660 (780)	420 (645)	570 (660)	600 (750)	600 (760)
Adherence to physical activity recommendation (yes) [*n* (%)]	408 (90.9)	320 (82.0)	302 (87.5)	255 (90.1)	181 (90.5)
Tumor stage [*n* (%)]					
Stage I	141 (30.7)	124 (31.3)	109 (31.3)	97 (33.8)	71 (34.1)
Stage II	108 (23.5)	100 (25.3)	86 (24.7)	69 (24.0)	52 (25.0)
Stage III	210 (45.8)	172 (43.4)	153 (44.0)	121 (42.2)	85 (40.9)
Cancer type [*n* (%)]					
Colon	290 (63.2)	250 (63.1)	222 (63.8)	181 (63.1)	126 (60.6)
Rectosigmoid and rectum	169 (36.8)	146 (36.9)	126 (36.2)	106 (36.9)	82 (39.4)
Treatment [*n* (%)]					
Surgery (yes)	412 (89.8)	354 (89.4)	317 (91.1)	259 (90.2)	186 (89.4)
Chemotherapy (yes)	184 (40.1)	155 (39.1)	134 (38.5)	107 (37.3)	79 (38.0)
Radiotherapy (yes)	116 (25.3)	101 (25.5)	88 (25.3)	73 (25.4)	55 (26.4)
Stoma (yes) [*n* (%)]	3 (0.7)	110 (28.4)	68 (19.8)	43 (15.2)	26 (13.1)

*BMI* body mass index, *MUAMC* mid-upper arm muscle circumference, *SD* standard deviation, *wk* week

^a^Percentages may not add to 100 due to rounding

^d^For accelerometer data *n* = 32

### Longitudinal associations of sedentary behavior, standing and physical activity with anthropometric measures

The coefficients presented in Table 2 present the overall, intra, and inter-individual longitudinal associations from 6 weeks to 24 months post CRC treatment. In the fully adjusted models, higher total and prolonged sedentary time were overall associated with lower handgrip strength (β per 2 h/day: -0.53 kg; 95% CI − 0.97, − 0.09 and − 0.22 kg; − 0.50, 0.05, respectively). No overall association was observed for MUAMC and measures of adiposity (BMI, waist circumference, and body fat). The intra-individual associations for total and prolonged sedentary time were statistically significant for handgrip strength (− 0.55 kg; − 1.06, − 0.05 and − 0.44 kg; − 0.79, − 0.09, respectively).

**Table 2:** Longitudinal associations of time spent on sedentary time, standing, light physical activity, and moderate-to-vigorous physical activity with body composition measures in colorectal cancer survivors

		BMI in kg**/**m^2^		Waist circumference in cm		Body fat in %		Mid-upper arm muscle circumference in cm		Handgrip strength in kg	
		β (95% CI)		β (95% CI)		β (95% CI)		β (95% CI)		β (95% CI)	
Total sedentary time (per 2 h/day)	Unadjusted	0.02	(− 0.14,0.17)	0.66*	(0.18,1.14)	− 0.05	(− 0.30,0.20)	0.36	(− 1.07,1.79)	− 0.32	(− 0.78,0.13)
	Adjusted^abe^	− 0.02	(− 0.15,0.10)	0.32	(− 0.09,0.74)	− 0.02	(− 0.23,0.19)	− 0.16	(− 1.53,1.21)	− 0.53*	(− 0.97,− 0.09)
	Intra^c^	− 0.03	(− 0.18,0.13)	0.29	(− 0.21,0.80)	− 0.06	(− 0.32,0.21)	− 0.55	(− 2.12,1.01)	− 0.55*	(− 1.06,− 0.05)
	Inter^d^	− 0.02	(− 0.20,0.16)	0.37	(− 0.25,1.00)	0.03	(− 0.27,0.33)	0.56	(− 1.20,2.32)	− 0.30	(− 0.91,0.30)
Prolonged sedentary time (per 2 h/day)	Unadjusted	− 0.04	(− 0.15,0.08)	0.20	(− 0.15,0.55)	− 0.05	(− 0.23,0.13)	− 0.47	(− 1.50,0.55)	− 0.59*	(− 0.91,− 0.26)
	Adjusted^abe^	− 0.02	(− 0.11,0.06)	0.14	(− 0.13,0.41)	0.00	(− 0.13,0.14)	− 0.20	(− 1.11,0.71)	− 0.22	(− 0.50,0.05)
	Intra^c^	− 0.05	(− 0.15,0.06)	0.10	(− 0.25,0.45)	− 0.07	(− 0.25,0.12)	− 0.28	(− 1.36,0.80)	− 0.44*	(− 0.79,− 0.09)
	Inter^d^	0.02	(− 0.10,0.14)	0.20	(− 0.21,0.60)	0.09	(− 0.11,0.30)	0.22	(− 0.96,1.40)	0.10	(− 0.32,0.51)
Standing (per hour/day)	Unadjusted	0.12*	(0.01,0.23)	− 0.08	(− 0.42,0.26)	0.15	(− 0.03,0.32)	0.17	(− 0.83,1.18)	0.31	(− 0.01,0.63)
	Adjusted^abe^	0.03	(− 0.05,0.12)	− 0.15	(− 0.42,0.12)	0.01	(− 0.13,0.15)	− 0.07	(− 0.90,0.75)	0.29*	(0.00,0.58)
	Intra^c^	0.05	(− 0.06,0.15)	− 0.14	(− 0.49,0.21)	− 0.01	(− 0.19,0.18)	0.43	(− 0.65,1.52)	0.36*	(0.01,0.71)
	Inter^d^	0.01	(− 0.11,0.14)	− 0.17	(− 0.58,0.24)	0.03	(− 0.17,0.23)	− 0.70	(− 1.88,0.49)	0.15	(− 0.26,0.56)
Light physical activity (per 8 h/day)	Unadjusted	0.06	(− 0.00,0.11)	0.01	(− 0.18,0.20)	0.12*	(0.02,0.22)	0.44	(− 0.14,1.01)	0.18	(− 0.01,0.36)
	Adjusted^abe^	0.02	(− 0.03,0.07)	0.03	(− 0.14,0.20)	0.03	(− 0.06,0.12)	0.20	(− 0.32,0.73)	0.23*	(0.07,0.40)
	Intra^c^	0.03	(− 0.03,0.08)	0.02	(− 0.17,0.21)	0.06	(− 0.04,0.16)	0.34	(− 0.26,0.95)	0.19	(− 0.01,0.39)
	Inter^d^	0.01	(− 0.10,0.11)	0.05	(− 0.30,0.40)	− 0.06	(− 0.23,0.12)	− 0.21	(− 1.26,0.83)	0.33	(− 0.02,0.68)
Moderate-to-vigorous physical activity (per 150 h/week)	Unadjusted	0.04*	(0.01,0.06)	0.05	(− 0.03,0.13)	0.03	(− 0.01,0.07)	0.40*	(0.15,0.64)	0.13*	(0.05,0.21)
	Adjusted^abe^	0.04*	(0.02,0.07)	0.07	(− 0.00,0.15)	0.07*	(0.03,0.10)	0.14	(− 0.11,0.39)	0.10*	(0.02,0.17)
	Intra^c^	0.04*	(0.01,0.07)	0.06	(− 0.03,0.15)	0.07*	(0.02,0.12)	0.14	(− 0.13,0.41)	0.07	(− 0.02,0.15)
	Inter^d^	0.05*	(0.01,0.10)	0.11	(− 0.04,0.25)	0.06	(− 0.01,0.13)	− 0.11	(− 0.53,0.30)	0.19*	(0.04,0.33)

*BMI* body mass index, *β* beta-coefficient, *CI* confidence interval, *cm* centimeter, *kg* kilogram

^a^Model adjusted for sex (male/female), age enrollment (years), co-morbidities (0, 1, ≥ 2), chemotherapy (yes/no), total energy intake (kcal·day^−1^), time since diagnosis (months), and respective anthropometric measure at diagnosis. Models for sedentary behavior were adjusted for moderate-to-vigorous physical activity (MVPA), and vice versa, and light-intensity physical activity was adjusted for MVPA. In addition, total sedentary time, prolonged sedentary time, and standing was adjusted for waking wear time (hours·day^−1^)

^b^The beta-coefficients represent the overall longitudinal difference in the outcome score

^c^The beta-coefficients represent the change in the outcome score over time within individuals

^d^The beta-coefficients represent the difference in the outcome score between individuals

^e^A random slope was added for the model MVPA with mid-upper arm muscle circumference; light physical activity with handgrip strength; standing with handgrip strength; total sedentary time with mid-upper arm muscle circumference and handgrip strength; prolonged sedentary time with BMI and mid-upper arm muscle circumference

^f^Prolonged sedentary time was the time accrued in uninterrupted sedentary bouts with a duration of at least 30 min

Accelerometer-assessed standing time was longitudinally associated with greater handgrip strength (β per hour/day: 0.29 kg; 95% CI 0.00, 0.58). This association appeared driven by an intra-individual component (0.36 kg; 0.01, 0.71). Similarly, higher self-reported LPA was longitudinally associated with greater handgrip strength (β per 8 h/week: 0.23 kg; 95% CI 0.07, 0.40). This association appeared to involve an intra-individual component (0.19 kg; − 0.01, 0.39) as well as an inter-individual component (0.33 kg; − 0.02, 0.68), although both were not statistically significant. Standing and LPA were not longitudinally associated with BMI, waist circumference, and body fat percentage.

Higher MVPA was statistically significantly associated with higher BMI (β per 150 min/week: 0.04 kg/m^2^; 95% CI 0.02, 0.07), higher body fat percentage (0.07%; 0.03, 0.10) and a greater handgrip strength (0.09 kg; 0.02, 0.17). All of these associations appeared to be driven by both the intra-individual and the inter-individual component. In particular, analyses of intra-individual associations showed that an increase of 150 min per week of MVPA within individuals over time was statistically significantly associated with a higher BMI (0.04 kg/m^2^; 0.01, 0.07) and higher body fat percentage (0.06%; 0.01, 0.11). The inter-individual analyses showed that individuals with 150 min per week higher average MVPA levels over time had statistically significantly higher BMI (0.05 kg/m^2^; 0.01, 0.10) and higher handgrip strength (0.35 kg; 0.08, 0.62) than individuals with lower MVPA levels.

In comparison to results of the main analysis, the overall associations of sedentary behavior and physical activity with body composition were attenuated in the time-lag analysis (Supplemental Table 1). Nevertheless, the directions of the associations were similar to associations based on analyses without the time-lag.

### Mediation analysis

**Fig. 1:**
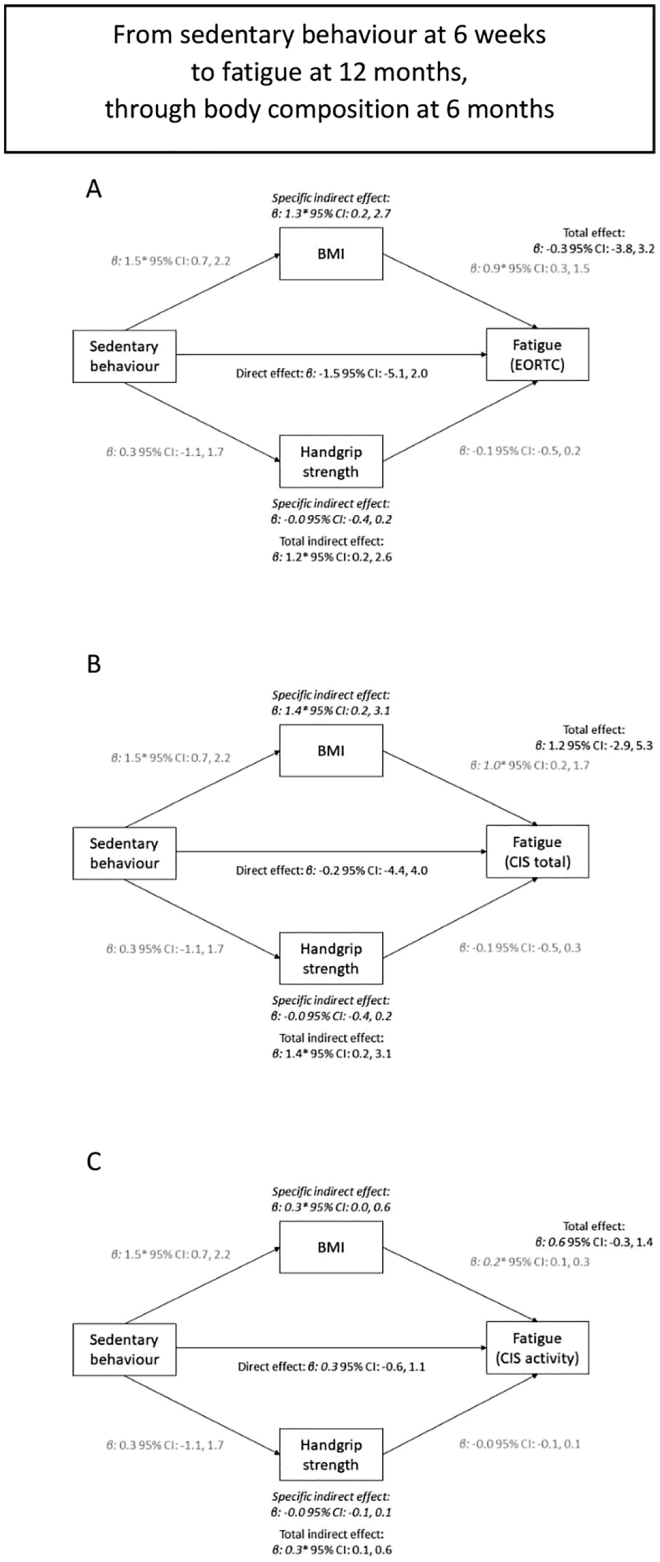
The association between total sedentary time at 6 weeks and fatigue at 12 months (total association), divided in a direct path independent of body composition at 6 months (direct association), and an indirect path via body composition (both BMI and handgrip strength – indirect association). Panel **A**, **B**, **C** used different questionnaires or subscales to assess fatigue (EORTC, CIS total, activity-related fatigue, respectively)

Mediation analysis between total sedentary time and fatigue showed that more sedentary time was associated with higher BMI and subsequently with more fatigue (Fig. 1 and Supplemental Fig. 1). The indirect specific association for BMI at 6 months was statistically significant for EORTC fatigue at 12 months (β per 2 h/day: 1.3; 95% CI 0.2, 2.7), total fatigue (CIS: 1.4; 0.2, 3.1), and activity-related fatigue (0.3; 0.0, 0.6), suggesting that part of the association between total sedentary time at 6 weeks and fatigue at 12 months was mediated by BMI at 6 months (Fig. 1). This specific indirect effect for BMI was in the same direction in the analyses of total sedentary time at 6 months and fatigue at 24 months, although not statistically significantly so (Supplemental Fig. 1). Handgrip strength did not play a mediating role in the association between sedentary time and fatigue (Fig. 1 and Supplemental Fig. 1).

**Fig. 2:**
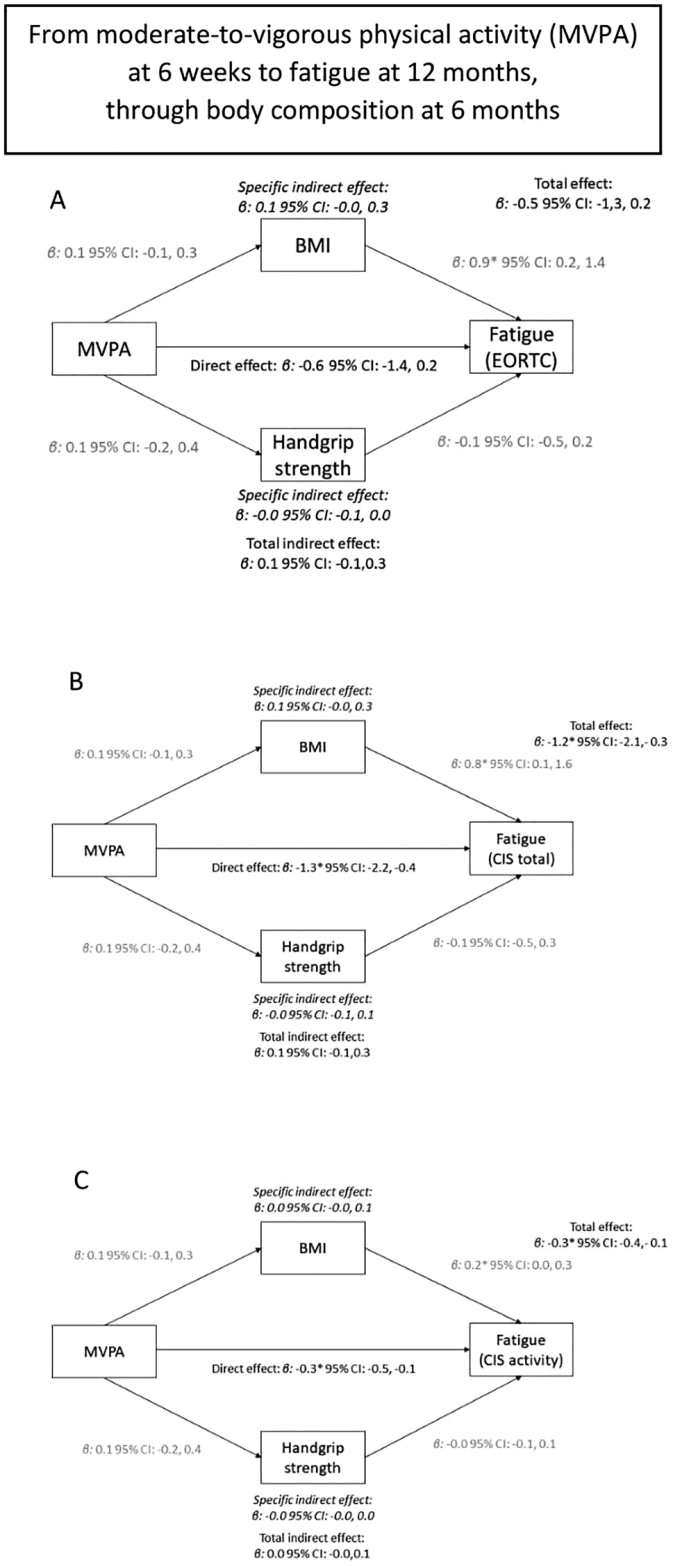
The association between moderate-to-vigorous physical activity at 6 weeks and fatigue at 12 months (total association), divided in a direct path independent of body composition (direct association), and an indirect path via body composition at 6 months (both BMI and handgrip strength—indirect association). Panel **A**, **B**, **C** used different questionnaires or subscales to assess fatigue (EORTC, CIS total, activity-related fatigue, respectively)

Mediation analysis showed total and direct associations between MVPA and fatigue, but no specific or total indirect associations through BMI or handgrip strength were observed (Fig. 2 and Supplemental Fig. 2).

Sensitivity analysis showed no differences between the 6 weeks to 12 months mediation analysis with a sample size of *n* = 230 and *n* = 170, except for wider confidence intervals (results not shown).

## Discussion

To our knowledge, this is the first study that assessed longitudinal associations of sedentary behavior, standing, LPA and MVPA with anthropometric measures in CRC survivors, from 6 weeks to 24 months post treatment. In addition, we also explored the role of body composition as a mediator in the association of sedentary behavior and MVPA with fatigue. In confounder-adjusted analyses, we observed that increased sedentary behavior, standing and LPA were associated with decreased handgrip strength, independent of MVPA, but not with measures of adipose tissue (BMI, waist circumference, and fat percentage). In addition, more MVPA was associated with greater adipose tissue and handgrip strength, independent of sedentary behavior. However, observed associations were small in general. We also observed that BMI, but not handgrip strength, may play a mediating role in the association between total sedentary time and fatigue, while no mediating role for these body composition variables was observed for the association between MVPA and fatigue.

Independent of MVPA, more total sedentary time, and less standing time and LPA was associated with decreased muscle mass and function, but not with measures of adipose tissue. No previous studies have studied longitudinal associations of objectively measured sedentary time or standing with body composition in CRC survivors. However, two published studies have characterized sedentary time in survivors of other types of cancer, using accelerometer data. These cross-sectional studies used data from the National Health and Nutrition Examination Survey (NHANES) (2003–2006). The first study also found positive associations of sedentary time with measures of adiposity (BMI, waist circumference and fasting insulin levels) in breast cancer survivors, yet these associations were weakened and non-significant when adjusting for MVPA (Lynch et al. 2010). The second study, however, found no statistically significant associations between sedentary time and adiposity in prostate cancer survivors with or without being adjusted for MVPA (Lynch et al. 2011).

In our study, mediation analysis showed that BMI could play a mediating role in the association between sedentary behavior and fatigue. Specifically, participants who spent more time in sedentary behavior experienced more fatigue approximately one year later, and a potential mechanism for this relation may involve increased BMI. The independent associations of increased sedentary time with increased BMI (Lynch et al. 2011) and of increased BMI with increased levels of fatigue have been described in the literature (Vissers et al. 2017; Neefjes et al. 2017), although not always consistent. To our knowledge, no study examined the mediating role of BMI in the association of sedentary behavior with fatigue to date.

Increased MVPA was statistically significantly associated with increased handgrip strength and, in contrast to what we expected, also with an increased BMI. This positive association was also observed for other adipose tissue outcomes, such as waist circumference and fat percentage, although results were not statistically significant. However, it should be noted that effect sizes were very small, which complicates the interpretability of these results. Two RCTs that assessed the relationship between exercise training and body composition in CRC survivors found decreases in (visceral) adipose tissue and increases in whole-body lean mass (Devin et al. 2016; Brown et al. 2017). A possible explanation for this observed difference and the small effect sizes that we observed is that we investigated habitual physical activity in a longitudinal study without intervention. In trials, people are encouraged to increase their physical activity and therefore greater changes and contrasts may be observed.

In a previous study conducted by our research group, we found that increased adipose tissue was associated with increased HRQoL and less fatigue (Kenkhuis et al. 2021a). We postulated that this could be due to a recovery of all aspects of body composition in the early post-treatment period. Both adipose tissue and muscle mass and function tend to decrease from diagnosis up to six weeks post treatment, and after that tend to increase up to 24 months post treatment, possibly indicating recovery from the impact of cancer treatment. This ‘recovery’ phase may also explain the longitudinal positive association found between MVPA and adipose tissue, which is likely to be bi-directional. Participants whose body composition (both adipose and lean body tissue) is recovering after the immediate cancer treatment phase are more likely to also show increasing MVPA or LPA levels in comparison to people who have not yet fully recovered.

Although mediation analysis supported the longitudinal association found between more MVPA and less fatigue, neither BMI nor handgrip strength was found to contribute as mediators of this association. In other words, more MVPA may lead to less fatigue, but possibly not through changes in adipose tissue and muscle mass and muscle function in the first two years post treatment.

A strength of our study included the use of objective accelerometer data, which enabled us to differentiate between sedentary behavior and standing, since these postures are fundamentally different physiologically (Berendsen et al. 2014; Chastin and Granat 2010). Furthermore, accelerometers provide ways to quantify measures of prolonged sedentary behavior, including prolonged sedentary time (Berendsen et al. 2014). Another strength of this study was the availability of extensive objective measures of adipose tissue, muscle mass and muscle function, although these measures are not considered the gold standard method for measuring body composition. Moreover, information on body composition was not obtained through self-report but collected by trained dietitians who performed anthropometric measurements according to strict measurement protocols, increasing the validity (Maukonen et al. 2018). Other strengths of our study included the high response rates during follow-up (> 90%), the limited number of missing data resulting from intensive data collection methods, and availability of extensive data on potential confounders. Although numbers decreased over time because participants had not yet reached all time points, mixed models is an analysis technique that efficiently deals with random missingness; the random missingness was confirmed by our sensitivity analyses.

There are also limitations that should be considered. Based on these observational data, we cannot be sure of the direction of associations of sedentary behavior and physical activity with body composition outcomes. Sedentary behavior, standing and physical activity may influence body composition, or the other way around, although our time-lag model did not show strongly attenuated associations supporting our hypothesis. A RCT is preferred to establish a causal effect. In addition, Bland–Altman plots indicated that the MOX-accelerometer had limited reliability for measuring physical activity at a moderate-to-vigorous intensity range (Berendsen et al. 2014); therefore, the activity monitor cannot accurately differentiate between LPA and MVPA. Nevertheless, the monitor enabled us to investigate the separate association of standing with body composition outcomes. For LPA and MVPA, questionnaire data were used resulting in potential reporting bias. For example, large differences can be seen when comparing the amount of self-reported LPA in this study to previous accelerometer-based research in cancer survivors (Lynch et al. 2013). In addition, the limited response rate at diagnosis (45%) might have resulted in selection bias. Participants with worse body composition and lower levels of physical activity and higher levels of sedentary behavior may have been less likely to participate, potentially having led to an attenuation of associations. Finally, we cannot rule out the possibility of false positive hypothesis tests due to the large number of tests performed.

In conclusion, this study showed that increased sedentary time, and decreased standing time and LPA were associated with increased muscle mass and function, but not with adiposity. Increased MVPA was associated with increased adipose tissue and muscle mass and functioning. Moreover, higher BMI, but not handgrip strength, may mediate the association between higher sedentary time and greater levels of fatigue. However, body composition did not play a mediating role in the association between higher MVPA and more fatigue. This study highlights that within the first years after diagnosis and treatment, changes of sedentary behavior, physical activity and body composition are interrelated. Future intervention studies should further investigate how body composition plays a mediating role within the association of sedentary behavior, standing, and physical activity with fatigue.

## Supplementary Information

Below is the link to the electronic supplementary material.

Supplementary file1 The association between total sedentary time at 6 months and fatigue at 24 months (total association), divided in a direct path independent of body composition at 12 months (direct association), and an indirect path via body composition (both BMI and handgrip strength – indirect association). Panel A,B,C used different questionnaires or subscales to assess fatigue (EORTC, CIS total, activity-related fatigue, respectively) (JPG 104 KB)

Supplementary file2 The association between moderate-to-vigorous physical activity at 6 months and fatigue at 24 months (total association), divided in a direct path independent of body composition (direct association), and an indirect path via body composition at 12 months (both BMI and handgrip strength - indirect association). Panel A,B,C used different questionnaires or subscales to assess fatigue (EORTC, CIS total, activity-related fatigue, respectively) (JPG 102 KB)

Supplementary file3 (DOCX 23 KB)

## Data Availability

Data described in the manuscript, code book, and analytic code will be made available upon request pending (e.g., application and approval, payment, other). Requests for data of the EnCoRe study can be sent to Dr. Martijn Bours, Department of Epidemiology, GROW School for Oncology and Reproduction, Maastricht University, the Netherlands (email: m.bours@maastrichtuniversity.nl).
