# Supplementary figures and images for: Longitudinal associations of sedentary behavior and physical activity with body composition in colorectal cancer survivors up to 2 years post treatment
Source: J Cancer Res Clin Oncol. 2022 Aug 30;149(7):4063–75. doi: 10.1007/s00432-022-04267-9 (PMC10314855; doi:10.1007/s00432-022-04267-9)

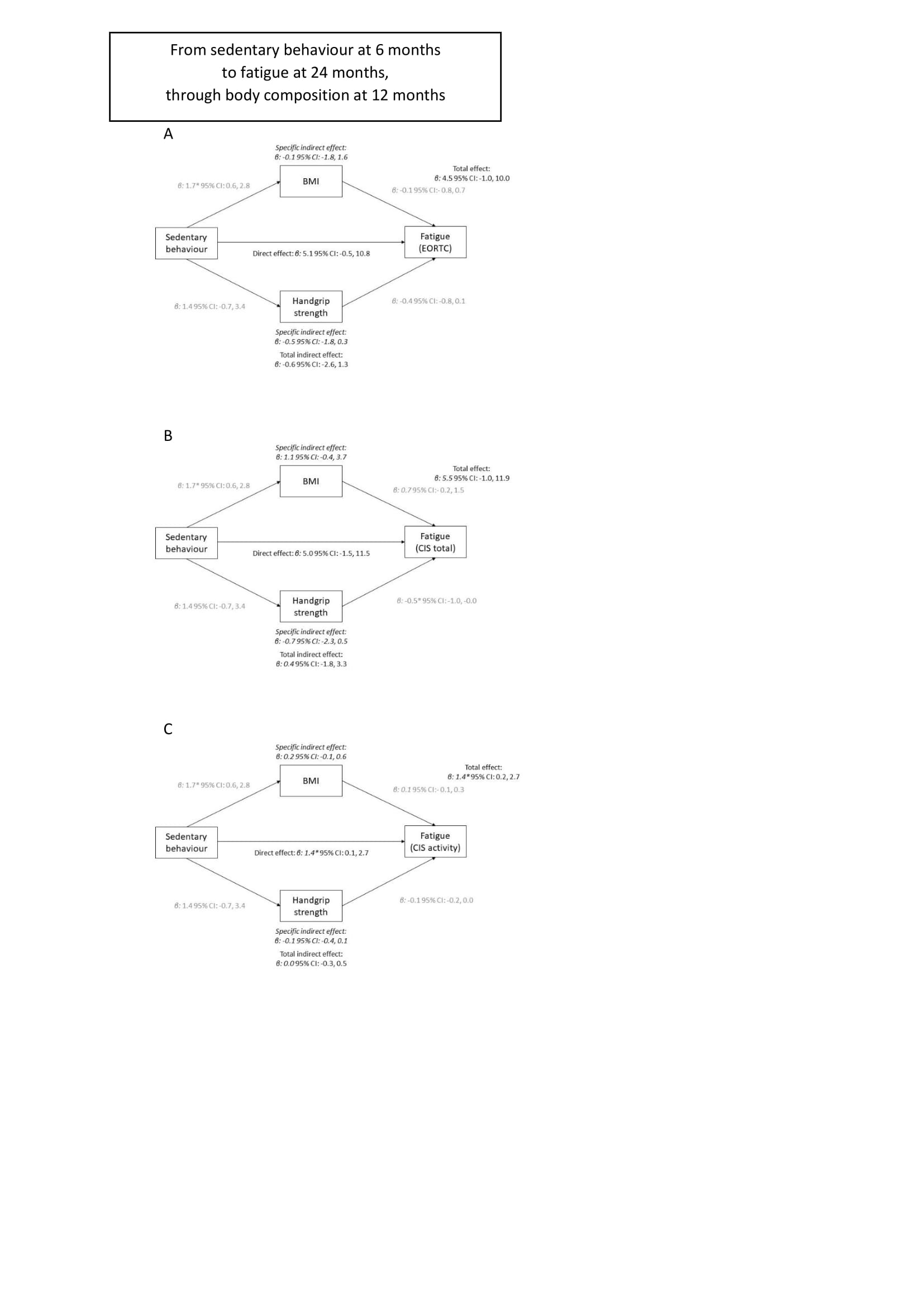

Supplement: Supplementary file 1 — Supplementary file1 The association between total sedentary time at 6 months and fatigue at 24 months (total association), divided in a direct path independent of body composition at 12 months (direct association), and an indirect path via body composition (both BMI and handgrip strength – indirect association). Panel A,B,C used different questionnaires or subscales to assess fatigue (EORTC, CIS total, activity-related fatigue, respectively) (JPG 104 KB) [file 432_2022_4267_MOESM1_ESM.jpg]

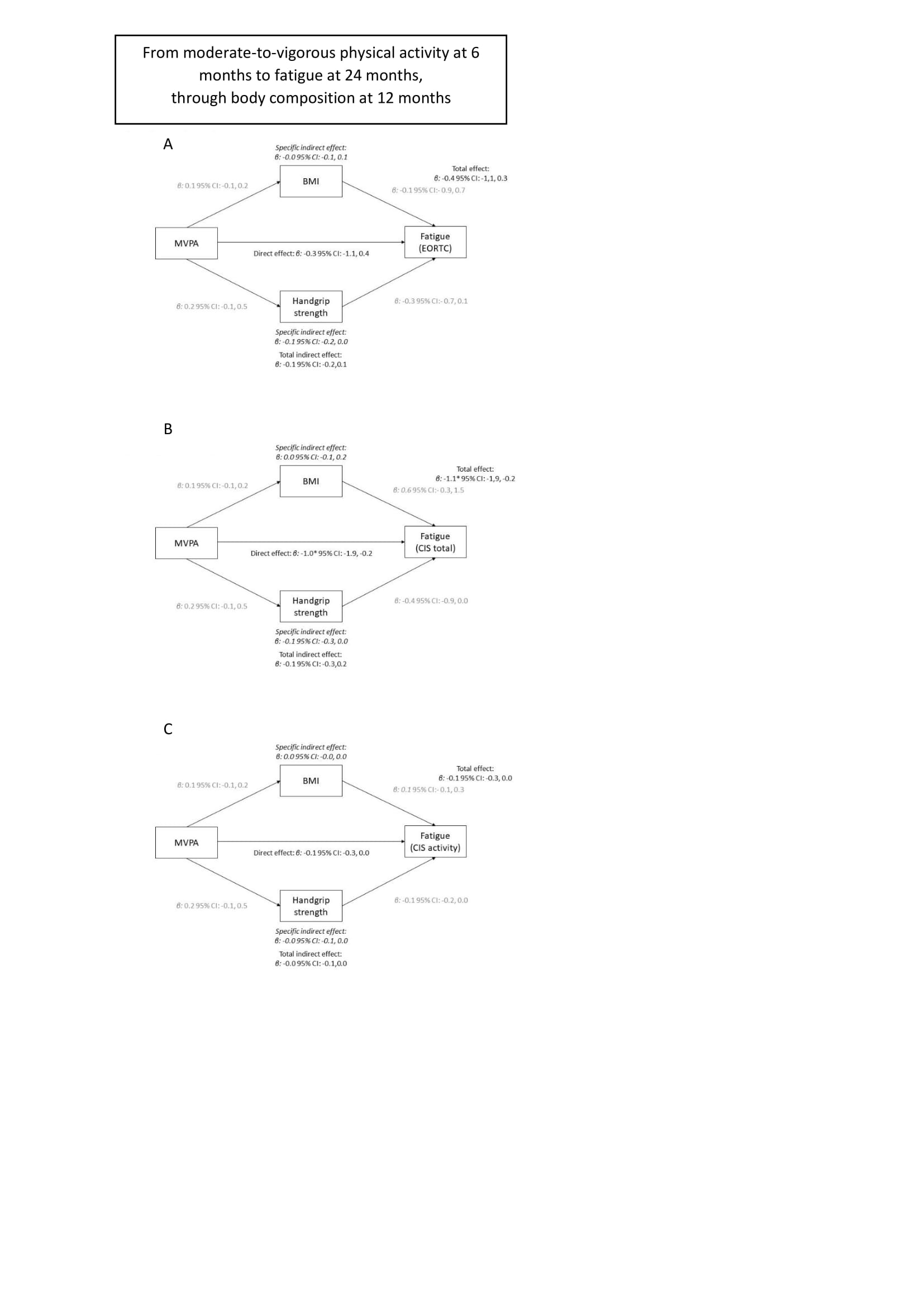

Supplement: Supplementary file 2 — Supplementary file2 The association between moderate-to-vigorous physical activity at 6 months and fatigue at 24 months (total association), divided in a direct path independent of body composition (direct association), and an indirect path via body composition at 12 months (both BMI and handgrip strength - indirect association). Panel A,B,C used different questionnaires or subscales to assess fatigue (EORTC, CIS total, activity-related fatigue, respectively) (JPG 102 KB) [file 432_2022_4267_MOESM2_ESM.jpg]
