# Supplementary material for: Longitudinal associations of sedentary behavior and physical activity with body composition in colorectal cancer survivors up to 2 years post treatment
Source: J Cancer Res Clin Oncol. 2022 Aug 30;149(7):4063–75. doi: 10.1007/s00432-022-04267-9 (PMC10314855; doi:10.1007/s00432-022-04267-9)
Supplement: Supplementary file 3 — Supplementary file3 (DOCX 23 KB) [file 432_2022_4267_MOESM3_ESM.docx]

Supplemental Table 1: Time-lag model: longitudinal associations of time spent in moderate-to-vigorous physical activity, light physical activity, standing and sedentary time with body composition measures in colorectal cancer survivors

|  | |  | BMI in kg**·**m**^-^**² | | Waist circumference  in cm | | Body fat in % | | Mid upper arm muscle circumference in cm | | Handgrip strength  in kg | |
| --- | --- | --- | --- | --- | --- | --- | --- | --- | --- | --- | --- | --- |
|  |  |  | β (95% CI) | | β (95% CI) | | β (95% CI) | | β (95% CI) | | β (95% CI) | |
| Total sedentary time  (per 2 hours·day^-1^) | | Unadjusted | 0.52* | (0.30,0.73) | 1.93* | (1.20,2.66) | 0.28 | (-0.08,0.65) | 3.09* | (0.86,5.33) | -0.28 | (-1.04,0.47) |
|  |  | Adjusted^abe^ | 0.11 | (-0.06,0.28) | 0.60* | (0.01,1.19) | 0.04 | (-0.25,0.33) | 0.81 | (-1.03,2.66) | -0.69* | (-1.33,-0.05) |
|  |  | Intra^c^ | 0.25* | (0.03,0.48) | 0.70 | (-0.10,1.50) | 0.10 | (-0.31,0.50) | -0.01 | (-2.65,2.63) | -0.89* | (-1.75,-0.03) |
|  |  | Inter^d^ | -0.05 | (-0.29,0.19) | 0.50 | (-0.31,1.31) | -0.02 | (-0.41,0.37) | 1.68 | (-0.46,3.82) | -0.30 | (-1.03,0.42) |
| Prolonged sedentary time^f^  (per 2 hours·day^-1^) | | Unadjusted | -0.05 | (-0.17,0.06) | -0.04 | (-0.45,0.36) | -0.13 | (-0.33,0.07) | -0.96 | (-2.24,0.33) | -0.46* | (-0.89,-0.03) |
|  |  | Adjusted^abe^ | -0.03 | (-0.14,0.07) | -0.02 | (-0.36,0.32) | -0.07 | (-0.24,0.10) | -0.64 | (-1.76,0.48) | -0.10 | (-0.46,0.25) |
|  |  | Intra^c^ | -0.09 | (-0.22,0.04) | -0.26 | (-0.73,0.21) | -0.17 | (-0.40,0.06) | -1.86* | (-3.43,-0.28) | -0.35 | (-0.86,0.16) |
|  |  | Inter^d^ | 0.07 | (-0.10,0.23) | 0.31 | (-0.25,0.97) | 0.06 | (-0.22,0.34) | 1.16 | (-0.40,2.72) | 0.17 | (-0.37,0.72) |
| Standing  (per hour·day^-1^) | | Unadjusted | -0.16* | (-0.30,-0.02) | -0.62* | (-1.10,-0.14) | -0.09 | (-0.32,0.15) | -1.63* | (-3.11,-0.16) | 0.09 | (-0.41,0.58) |
|  |  | Adjusted^abe^ | -0.05 | (-0.16,0.06) | -0.28 | (-0.65,0.10) | -0.04 | (-0.23,0.14) | -0.78 | (-1.90,0.33) | 0.21 | (-0.19,0.60) |
|  |  | Intra^c^ | -0.11 | (-0.25,0.03) | -0.28 | (-0.79,0.23) | -0.20 | (-0.45,0.06) | 0.44 | (-1.23,2.11) | 0.35 | (-0.19,0.90) |
|  |  | Inter^d^ | 0.03 | (-0.13,0.19) | -0.27 | (-0.81,0.26) | 0.12 | (-0.14,0.38) | -1.67* | (-3.10,-0.23) | 0.13 | (-0.36,0.62) |
| Light physical activity (per 8 hours·day^-1^) | | Unadjusted | 0.06 | (-0.01,0.12) | -0.02 | (-0.24,0.20) | 0.16* | (0.04,0.27) | 0.13 | (-0.56,0.82) | 0.19 | (-0.04,0.41) |
|  |  | Adjusted^ab^ | 0.02 | (-0.04,0.07) | -0.04 | (-0.24,0.16) | 0.03 | (-0.07,0.14) | 0.25 | (-0.40,0.89) | 0.28* | (0.07,0.48) |
|  |  | Intra^c^ | 0.03 | (-0.03,0.10) | -0.03 | (-0.25,0.19) | 0.07 | (-0.04,0.19) | 0.38 | (-0.36,1.12) | 0.27* | (0.03,0.51) |
|  |  | Inter^d^ | -0.05 | (-0.17,0.08) | -0.10 | (-0.51,0.32) | -0.09 | (-0.30,0.11) | -0.15 | (-1.39,1.12) | 0.30 | (-0.09,0.70) |
| Moderate-to-vigorous physical activity  (per 150 hours·week^-1^) | | Unadjusted | 0.05* | (0.02,0.07) | 0.12* | (0.03,0.21) | 0.04 | (-0.01,0.09) | 0.41* | (0.12,0.70) | 0.17* | (0.08,0.27) |
|  |  | Adjusted^ab^ | 0.04* | (0.01,0.06) | 0.08 | (-0.01,0.17) | 0.08* | (0.03,0.12) | -0.04 | (-0.32,0.25) | 0.11* | (0.02,0.20) |
|  |  | Intra^c^ | 0.04* | (0.01,0.07) | 0.10 | (-0.01,0.20) | 0.07* | (0.02,0.12) | -0.03 | (-0.37,0.31) | 0.11 | (-0.01,0.22) |
|  |  | Inter^d^ | 0.04 | (-0.01,0.10) | 0.03 | (-0.15,0.21) | 0.09* | (0.00,0.18) | -0.05 | (-0.55,0.45) | 0.12 | (-0.05,0.29) |

Abbreviations: BMI, body mass index; β, beta-coefficient; CI, confidence interval; cm, centimeter; kg, kilogram

^a^ Model adjusted for sex (male/female), age enrolment (years), co-morbidities (0, 1, ≥2), chemotherapy (yes/no), total energy intake (kcal·day^-1^), time since diagnosis (months), and respective anthropometric measure at diagnosis. Models for sedentary behavior were adjusted for moderate-to-vigorous physical activity (MVPA), and vice versa, and light-intensity physical activity was adjusted for MVPA. In addition, total sedentary time, prolonged sedentary time, and standing was adjusted for waking wear time (hours·day^-1^).

^b^ The beta-coefficients represent the overall longitudinal difference in the outcome score.

^c^ The beta-coefficients represent the change in the outcome score over time within individuals.
^d^ The beta-coefficients represent the difference in the outcome score between individuals.

^e^ A random slope was added for the model MVPA with mid upper arm muscle circumference; light physical activity with handgrip strength; standing with handgrip strength; total sedentary time with mid upper arm muscle circumference and handgrip strength; prolonged sedentary time with BMI and mid upper arm muscle circumference.
^f^ Prolonged sedentary time was the time accrued in uninterrupted sedentary bouts with a duration of at least 30 minutes
